# Recruiting Community Partners for Veggie Van: Strategies and Lessons Learned From a Mobile Market Intervention in North Carolina, 2012–2015

**DOI:** 10.5888/pcd14.160475

**Published:** 2017-04-27

**Authors:** Gina L. Tripicchio, Jacqueline Grady Smith, Janelle Armstrong-Brown, Jared McGuirt, Lindsey Haynes-Maslow, Sarah Mardovich, Alice S. Ammerman, Lucia Leone

**Affiliations:** 1 Department of Nutrition, Gillings School of Global Public Health, University of North Carolina at Chapel Hill, Chapel Hill, North Carolina; 2 Center for Health Promotion and Disease Prevention, University of North Carolina at Chapel Hill, Chapel Hill, North Carolina; 3 RTI International, Research Triangle Park, North Carolina; 4 Department of Agricultural and Human Sciences, North Carolina State University, Raleigh, North Carolina; 5 Department of Community Health and Health Behavior, University at Buffalo-SUNY, Buffalo, New York.

## Abstract

**Background:**

Food access interventions are promising strategies for improving dietary intake, which is associated with better health. However, studies examining the relationship between food access and intake are limited to observational designs, indicating a need for more rigorous approaches. The Veggie Van (VV) program was a cluster-randomized intervention designed to address the gap between food access and intake. In this article, we aim to describe the approaches involved in recruiting community partners to participate in VV.

**Community Context:**

The VV mobile market aimed to improve access to fresh fruits and vegetables by providing subsidized, high-quality, local produce in low-resource communities in North Carolina. This study describes the strategies and considerations involved in recruiting community partners and individual participants for participation in the VV program and evaluation.

**Methods:**

To recruit partners, we used various strategies, including a site screener to identify potential partners, interest forms to gauge future VV use and prioritize enrollment of a high-need population, marketing materials to promote VV, site liaisons to coordinate community outreach, and a memorandum of understanding between all invested parties.

**Outcome:**

A total of 53 community organizations and 725 participants were approached for recruitment. Ultimately, 12 sites and 201 participants were enrolled. Enrollment took 38 months, but our approaches helped successfully recruit a low-income, low-access population. The process took longer than anticipated, and funding constraints prevented certain strategies from being implemented.

**Interpretation:**

Recruiting community partners and members for participation in a multi-level, community-based intervention was challenging. Strategies and lessons learned can inform future studies.

## Background

Improving dietary intake is associated with better health (1), and it is imperative for research efforts to promote strategies that decrease diet-related disease prevalence (2). High-need populations have substantially lower intake of fruits and vegetables (3), and are disproportionately affected by negative health outcomes associated with poor dietary intake including hypertension (4), cardiovascular disease (5), obesity (6), and risk of some cancers (7). Although there may be many reasons for poor diets, lack of access, preparation time, knowledge, and transportation are common barriers (8). Therefore, interventions aiming to improve access to affordable, healthy food might be a promising strategy for improving health in these populations (9,10). Understanding the relationship between access to healthy food and intake is important, but most studies are limited to cross-sectional observational data (11) and literature on the effect of interventions to modify access and intake is generally lacking (12). The Veggie Van (VV) program was designed to address this gap and evaluate a food access program using a rigorous multilevel, randomized design.

To reach communities with limited access, the VV program aimed to establish partnerships with community organizations for the intervention. “Limited access” refers to communities in which high-quality, affordable, fresh fruits and vegetables are not readily available. Community sites, such as schools, faith-based organizations, health clinics, worksites and community centers, are effective places to reach groups of people for health interventions (13). Community leaders have institutional knowledge and expertise in working with priority populations (14), and their involvement can help cultivate community members’ trust (15). Despite the necessity of community engagement in recruitment processes, the strategies, challenges, and considerations involved in recruiting community partners and individual study participants are underreported. Thus, this study aims to describe the approaches involved in recruiting community partners and individual study participants for a cluster-randomized food access intervention.

## Community Context

The VV program was implemented in collaboration with organizations in communities in North Carolina that lack access to affordable, fresh fruits and vegetables. Organizations serving low-income, diverse populations were identified as the priority for recruitment because of significantly lower rates of fruit and vegetable consumption among these groups in North Carolina. According to the most recent report, only 15.2% of low-income adults in North Carolina (ie, annual household income of ≤$15,000) consume 5 or more servings of fruits and vegetables per day, compared with 25.0% of adults who make $50,000 or more (16). The goal of VV was to improve fruit and vegetable access and intake among participants at partner organizations receiving the intervention. The purpose of our community engagement efforts was to 1) enroll community organizations to receive the VV mobile market program and participate in the study, and 2) identify potential VV customers and recruit them to participate in the study.

## Methods

The VV program was a mobile market, which provided local North Carolina produce to community sites at a subsidized cost and also aimed to provide nutrition education, cooking demonstrations, and seasonal recipes (17). “Mobile market” generally refers to the selling of fresh produce via portable means. The VV mobile market sold produce shares, similar to a community-supported agriculture program, as well as individual items, and was operated once per week at each community site. Before starting VV, members of low-income communities participated in focus groups and provided information on barriers and perceptions related to fruit and vegetable access in their communities (8,18). This information was used to guide the development of the VV program, and community partners were engaged in the implementation of the VV mobile market at their specific location.

The study was a randomized controlled trial designed to assess the impact of VV on individual fruit and vegetable access and consumption. The recruitment team, which consisted of study researchers and program staff, searched for potential partners through established community relationships and by identifying organizations that served the target population and met study criteria. Potential partners were eligible to participate if they 1) had a person willing to act as a site liaison, 2) were willing to be randomized as part of the research study, 3) could recruit at least 30 interested community members to participate in the study, and 4) had space to accommodate the VV mobile market weekly (indoor and outdoor). Site liaisons were responsible for promoting the VV program, coordinating recruitment efforts, and acting as the support for the duration of the program. A description of the site liaison roles and their community organization are presented in Table 1.

**Table 1:** Characteristics of Study Sites and Corresponding Site Liaison Roles, Enrollment Rates, and Recruitment Timelines, Veggie Van Mobile Market Intervention, North Carolina, 2012–2015

Site	Type of Organization	Site Liaison Role	Interest Form Enrollment Rate, %	Recruitment Time^a^, Months
1	Recreation center	Employee	19.2	5.6
2		Employee	41.3	4.3
3		Employee	26.7	12.6
4	Housing community	Volunteer/after school coordinator	50.0	7.7
5	Health department/health clinic	Employee	34.6	9.5
6		Employee	26.1	9.7
7		Employee	35.7	9.4
8		Employee	28.8	16.3
9	Community center	Director/community leader	18.2	22.5
10		Director/community leader	19.6	15.4
11	Public library	Employee	35.2	5.9
12	Nonprofit community organization	Employee	25.7	4.9

a Recruitment time indicates the total time it took to recruit the site and the participants.

A screener questionnaire was developed to determine whether potential partners could effectively reach and engage community members. The screener provided information on 1) programming that attracted people to the location on a regular basis (eg, classes, meetings, events), 2) strategies for communication (eg, newsletters, websites), 3) feasibility of offering VV at a site year-round, and 4) the possibility of reaching a significant number of people. We ultimately wanted to collaborate with organizations that could meet study goals but also sustain the VV program after study completion. The goal was to recruit 12 community partner organizations and 25 participants from each site. This goal was estimated based on power calculations to detect change in daily fruit and vegetable intake between the intervention and control groups.

After screening potential partners for eligibility, forms were collected to assess interest among individuals in the community. For potential partners to become a study site, a minimum of 30 community members needed to express interest in using VV. The first question on the interest form asked potential participants about their interest in using a mobile market program. Only those who were interested were contacted for study enrollment. This was important in a pilot study because the research team could not test efficacy of the program if no one in the study actually used the program. We also asked about receipt of government assistance (eg, Supplemental Nutrition Assistance Program [SNAP], Special Supplemental Nutrition Program for Women, Infants, and Children [WIC]), barriers to fruit and vegetable intake (eg, cost, access, time to prepare), number of children in the household, and weekly spending on fruits and vegetables. The forms were available in both Spanish and English, but participants needed to be able to write and speak in English to be eligible for the study. Therefore, only participants who completed forms in English were contacted for study enrollment.

Various strategies were used to inform participants about the program and collect interest forms. First, marketing materials were created and efforts were made to create transparent, informative communication. Materials were available in both English and Spanish. The marketing materials were designed to recruit study participants, but they were also used to promote use of the VV mobile market program. Therefore, we wanted materials to be accessible to both English- and Spanish-speaking populations. Sites were diverse in the populations they served, and most requested materials in different formats (eg, brochures, flyers, posters). The recruitment team participated in special community events and alongside regularly scheduled activities where they conducted cooking demonstrations, distributed healthy recipes, collected interest forms, and answered questions about VV. Community partners often organized the special community events (eg, Unity in the Community event, annual fundraisers) and invited VV to attend as a community partner. Materials were also distributed through web-based platforms (eg, email, Facebook, Twitter) and by posting information on bulletin boards and in community newsletters. Community engagement efforts were time intensive, so a part-time recruitment coordinator was hired to manage the outreach and communication efforts between the recruitment team and the potential community partners. Having a coordinator to maintain communications, develop materials, and oversee timelines was essential to community-based recruitment.

After enough interest forms were collected, a memorandum of understanding (MOU) was signed and the information on the forms was used to contact and enroll study participants. Community sites were then matched as pairs and randomized to the intervention or delayed intervention control group. All participants provided informed consent, and the institutional review board at the University of North Carolina at Chapel Hill approved the study.

## Outcome

Recruitment of community partners and participants for a site-randomized trial was challenging. The process took more time and resources than we originally anticipated on the basis of our formative research. Each site required a tailored approach, and developing consistent branding, messaging, and information for use across multiple community sites was challenging. Adjustments and tradeoffs were made to balance enrollment goals and study timelines. Lessons learned about community and participant recruitment strategies are described in more detail below and in Table 2.

**Table 2:** Recruitment Strategies, Issues and Challenges Encountered, and Guidance for Future Researchers, Veggie Van Mobile Market Intervention, North Carolina, 2012–2015

Recruitment Strategy	Description	Level of Recruitment	Issues/Challenges Encountered	Guidance for Future Investigators
Site screener	A screener questionnaire used to determine if sites could meet goals for community engagement and study implementation	Community site	The screener was not as useful as intended for identifying potential partners; use of the screener was discontinued half-way through the study, and site liaisons were prioritized as a more promising strategy for getting sites enrolled.	Another strategy for identifying potential partners would be more effective than a screener questionnaire.
Site liaison	A person within a community organization designated as the main point of contact for the recruitment, VV program, and research teams	Community site	Site liaisons were volunteers or employees who worked directly with our priority population but were not always members of the community themselves; motivated site liaisons were able to expedite requirements for site recruitment, but all site liaisons had difficulty engaging community members.	Future studies should consider hiring a community member to act as liaison for engagement and recruitment.
Marketing materials	Materials created to advertise the VV program	Community sites and participants	Marketing materials needed to be customized based on the audience (ie, community partners and study participants); community partners had different outlets for relaying information to members including brochures, flyers, email, online platforms, and in person meetings.	Identify preferred languages of target communities and ensure that there are resources for creating materials for all audiences; create a common marketing message and create consistent program branding regardless of advertising outlet.
Interest forms	Forms used to screen participant eligibility, gauge interest in the VV program, prioritize enrollment of high-need participants, and obtain consent for study participation	Participant	On average, every 4 forms collected yielded 1 enrolled study participant.	The use of forms to prioritize enrollment did help us successfully enroll a low-income, low-access sample; online forms were used and could provide additional recruitment success in future studies.
Memorandum of Understanding (MOU)	An agreement of terms signed between the community partner organization, the VV program team, and the research team	Community site	The execution of the MOU took much longer than desired at some sites because of bureaucratic issues.	All sites enrolled in the study signed the MOU; this was a necessary entity, especially for organizations that required board or legal approval of their own.
Block enrollment	Participants’ self-reporting receipt of government assistance was prioritized for recruitment first, followed by those self-reporting barriers to fruit and vegetable intake. Participants who did not report either were recruited last.	Participant	Because of an overall lack of forms, eventually all eligible potential participants were contacted, regardless of block.	The block enrollment approach helped us to successfully enroll a low-income, low-access population, but this also could have been a result of our targeted recruitment efforts since all participants who completed forms eventually needed to be contacted for enrollment.

Abbreviation: VV, Veggie Van.

### Community site recruitment

**Figure 1:**
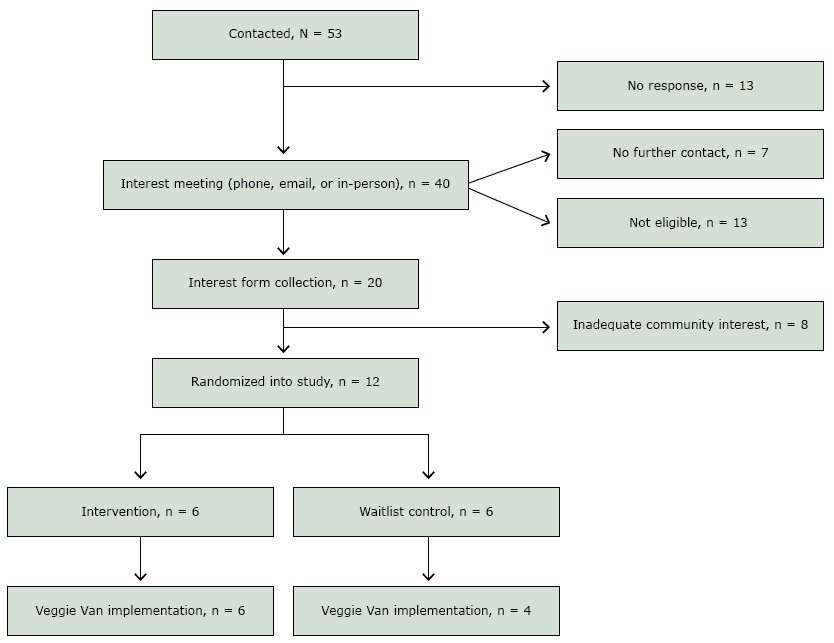
Recruitment and randomization of community sites, Veggie Van Mobile Market Intervention, North Carolina, 2012–2015.

A total of 53 sites were initially contacted; 40 responded and met with the recruitment team to assess interest and program fit (Figure 1). Communications occurred via telephone, email, and in-person meetings. From the 40 contacted, 7 sites became unresponsive, and 13 sites were not eligible to move forward because they could not identify a person to designate as the site liaison, lacked adequate indoor or outdoor space to accommodate VV, or did not have regularly scheduled events that brought community members to their location. All organizations were contacted at least twice. Although there were no set standards for how many contacts were made before designating a site unresponsive, we concluded that sites would probably not be able to sustain the communication needed to have a program partnership if they were not reachable or did not respond after initial contacts were made. Twenty organizations were deemed eligible and initiated the process to gauge support among the community, but only 12 were able to obtain adequate potential participant interest (ie, at least 30 interest forms from the priority population). The final community partner organizations included recreation centers, a housing community, health departments or clinics, community centers, a public library, and a nonprofit community organization (Table 1). The organizations that were eligible but did not collect enough interest forms were mainly faith-based organizations and childcare centers. These organizations had access to smaller communities and were overburdened, making it a challenge to provide additional programs and services. However, leaders of faith-based and childcare centers can cultivate consistent and strong relationships with community members, and future studies should consider ways in which researchers could alleviate potential burdens on such organizations to broker more efficient recruitment (19).

The randomized study design added complexities to the entire recruitment process. Potential partners had difficulty understanding why there was a research component associated with the program. Others were resistant to the idea of being a control site, and others perceived timelines associated with research as a barrier to participation. Providing research training for site liaisons could have provided transparency and made partners feel more engaged in both the program and research components. Additionally, matching sites for randomization resulted in delays between assessing interest and enrolling participants into the study. We could have randomized community sites before enrolling participants, but we would have risked low enrollment and threats to study validity. Also, a goal of the study was to meet the needs of the community and its members, and therefore buy-in on both levels (individual and community) was important before initiating the program. Collecting qualitative data on why potential sites and participants were not interested in the study could have provided more insight. Anecdotally, it was very challenging to get people interested in a program that did not yet exist, and generally people seemed resistant to the idea of research.

**Figure 2:**
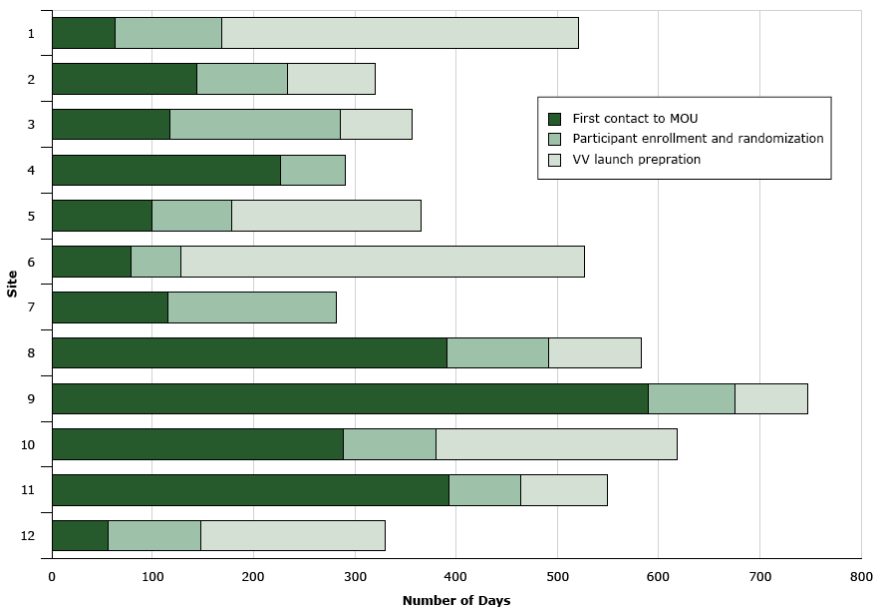
Timelines associated with each phase of site recruitment, by site number, Veggie Van Mobile Market Intervention, North Carolina, 2012–2015. Abbreviations: MOU, memorandum of understanding; NA, not applicable. **Table:** 

In total, site recruitment for this 12-site RCT took 38 months. Recruitment started in August 2012, and all sites were successfully recruited and randomized by October 2015. Site descriptions and recruitment characteristics are presented in Table 1. Across 12 study sites, individual recruitment ranged from 4.2 to 22.5 months, with an average of 10 months per site. Partner organizations were highly variable in the time each phase of recruitment lasted (Figure 2).

Site	First Contact to MOU	Participant Enrollment and Randomizaiton	VV Launch Prepration
	No. of Days		
1	62	105	353
2	143	89	87
3	116	169	70
4	226	64	NA
5	99	78	187
6	78	49	399
7	114	167	NA
8	390	100	92
9	589	85	72
10	288	91	238
11	392	71	85
12	55	92	182

Administrative and organizational issues, specifically those associated with the execution of the MOU, hindered the recruitment process at some sites. Although recruitment materials were explicit and meetings were organized to expedite this process, busy schedules and organizational hierarchy slowed the process significantly. The screener questionnaire was also not as helpful at identifying potential partners as anticipated, and we eventually stopped using it. Site liaisons seemed to facilitate recruitment efforts, and we made finding motivated, engaged site liaisons a priority.

Although site liaisons facilitated overall recruitment processes at some sites, they varied in their interaction and reach within communities. Unfortunately, in this study, funding to incentivize the site liaison or hire a champion directly from the community was not possible. Therefore, the volunteer site liaisons in this study were mostly employees of organizations that worked directly with high-need communities, but some were not members of those communities themselves. The site liaisons that did interact with community members in a regular, ongoing capacity expedited interest form collection and improved timelines for program implementation. Given this, hiring a community champion or creating a community advisory board could have facilitated community outreach if resources were available. A core principle of community-based participatory research is equitable collaboration and the integration of community members, and paid staff would not only have bolstered recruitment but could have improved overall community engagement and program success (20). In this study, it was important to approach all relationships carefully to avoid making promises to vulnerable communities that might not be upheld if partnerships were not solidified. The need for community engagement had to be balanced with timelines and funding, and trust and transparency around research protocols and processes needed to be fostered. Again, putting more resources into hiring community members to foster trust might have improved our success.

### Participant recruitment

A total of 725 interest forms were collected and served multiple purposes including screening community members to gauge interest in VV, obtaining consent for study participation, and collecting data to prioritize recruitment of high-need populations. Most interest forms were collected directly by site liaisons; attending community events (special one-time events and regularly scheduled events) was the least effective strategy. We were hopeful that attending these events would demonstrate investment in partner organizations and provide an opportunity to share information with potential study participants, in person. However, given that these events required staff, volunteers, and materials, there was little benefit when only a few community members were engaged or completed forms.

Language was also a barrier, as we received a fair amount of interest forms in Spanish. Unfortunately, we did not have the program staff or research approval to include Spanish-speaking participants in the study and this could have improved recruitment efforts. Surprisingly, online interest forms were more successful than anticipated. We did not think online forms would be effective for reaching low-income, low-access populations. However, we added online forms as an option to decrease the time between interest form completion and enrollment. Community members from 6 of the 12 sites used the online form and had a higher enrollment rate than the in-person forms. Additionally, online forms eliminated the potential for illegible handwriting and incomplete forms, which were issues with the handwritten forms.

From the initial forms (N = 725), 71.2% (n = 516) indicated an interest in participating in VV and were contacted for enrollment. Reaching participants by telephone presented some challenges; often telephone numbers were incorrect or disconnected. Ultimately, a little more than half (58.7%, n = 303) of potential participants were reachable by telephone; of those, 96.0% (n = 291) were eligible on the basis of inclusion criteria. To improve enrollment, we could have considered implementing in-person eligibility screenings where participants were consented, enrolled, and scheduled for a survey at the time of interest form completion. Although this approach may have improved recruitment and facilitated reliable data collection, it is time- and resource-intensive and perhaps not realistic for most large randomized trials.

After initial eligibility and screening, 90 participants became unreachable and did not complete the baseline survey. The final baseline study sample included 201 participants, indicating that, on average, for every 4 interest forms collected, 1 participant was enrolled. To prioritize enrollment of a low-income, low-access population, participants were categorized into blocks on the basis of self-report of 1) receipt of government assistance, 2) barriers to fruit and vegetable intake (but no government assistance), and 3) no receipt of government assistance or barriers. Recruitment rates by priority group are shown in Table 3. Of all interested potential participants (N = 516), most (50.8%, n = 262) reported receiving government assistance, followed by those who reported barriers to fruit and vegetable intake (25.6%, n = 132) and those reporting neither (19.6%, n = 101). A small percentage of participants (4%, n = 21) were not classified at all. Assigning potential participants to blocks for recruitment did help successfully enroll a low-income, low-access priority population.

**Table 3:** Rates of Participant Baseline Recruitment, by Priority Block, Veggie Van Mobile Market Intervention, North Carolina, 2012–2015

Block^a^	All (N = 516)	Enrolled (n = 201)	% Enrolled
Government assistance	262	95	36.3
Barriers to fruit and vegetable intake	132	53	40.2
No government assistance or barriers to fruit and vegetable intake	101	39	38.6
No block identified	21	14	66.7

a To prioritize enrollment of a low-income, low-access population, participants were categorized into blocks based on self-report of 1) receipt of government assistance, 2) barriers to fruit and vegetable intake (but no government assistance), and 3) no receipt of government assistance or barriers.

## Interpretation

Despite the difficulties discussed, we successfully recruited low-income, low-access participants for a food access intervention in North Carolina and learned lessons that can help future community-based trials. Strategies included engaging and supporting community partners, using unique strategies such as interest forms, and maintaining communication throughout the recruitment process. Hiring a site recruitment coordinator to organize recruitment efforts was essential. However, hiring community members as part of the research team may also have increased participant recruitment and decreased recruitment time.

Funding organizations should allow researchers adequate time to engage in community outreach and provide sufficient funding to support the contributions of community members who can act as liaisons, because their help is critical in building equity in community-based research. Additionally, smaller funding mechanisms could be helpful for supporting the initiation of community–academic partnerships, and larger funding mechanisms should have more realistic expectations for recruitment and engagement timelines for community-based studies.

Clearly communicating timelines and expectations, establishing strong community partnerships, and implementing effective recruitment strategies are critical for program success. Despite more rigorous outcomes, a randomized design could have deterred the willingness and investment of community partners. Thus, future studies should aim to expand literature on this topic, especially for multilevel, randomized, community-based trials. By presenting successes, challenges, and timelines associated with this intervention, we are hopeful that our findings may be beneficial for both investigators and community health practitioners aiming to implement community-based food access initiatives.
